# Delayed oseltamivir plus sirolimus treatment attenuates H1N1 virus-induced severe lung injury correlated with repressed NLRP3 inflammasome activation and inflammatory cell infiltration

**DOI:** 10.1371/journal.ppat.1007428

**Published:** 2018-11-13

**Authors:** Xuehong Jia, Bo Liu, Linlin Bao, Qi Lv, Fengdi Li, Hui Li, Yunqing An, Xulong Zhang, Bin Cao, Chen Wang

**Affiliations:** 1 Peking University China-Japan Friendship School of Clinical Medicine, Beijing, China; China-Japan Friendship Hospital, Capital Medical University, Beijing, China; 2 Department of Pulmonary and Critical Care Medicine, Department of Clinical Microbiology, Zibo City Key Laboratory of Respiratory Infection and Clinical Microbiology, Linzi District People’s Hospital, Zibo, Shandong, China; 3 Institute of Laboratory Animal Sciences, Chinese Academy of Medical Sciences (CAMS) and Comparative Medicine Center, Peking Union Medical College (PUMC), Key Laboratory of Human Disease Comparative Medicine, Ministry of Health, Beijing, China; 4 Department of Pulmonary and Critical Care Medicine, Center for Respiratory Diseases, China-Japan Friendship Hospital, Beijing, China; 5 Department of Immunology, School of Basic Medical Sciences, Capital Medical University, Beijing, China; 6 Clinical Center for Pulmonary Infections, Capital Medical University, Beijing, China; 7 National Clinical Research Center for Respiratory Diseases, Beijing, China; 8 Tsinghua University-Peking University Joint Center for Life Sciences, Beijing, China; 9 Chinese Academy of Medical Sciences (CAMS), Peking Union Medical College (PUMC), Beijing, China; 10 Beijing Key Laboratory of Respiratory and Pulmonary Circulation Disorders, Beijing, China; St. Jude Children's Research Hospital, UNITED STATES

## Abstract

Severe influenza A virus infection causes high mortality and morbidity worldwide due to delayed antiviral treatment and inducing overwhelming immune responses, which contribute to immunopathological lung injury. Sirolimus, an inhibitor of mammalian target of rapamycin (mTOR), was effective in improving clinical outcomes in patients with severe H1N1 infection; however, the mechanisms by which it attenuates acute lung injury have not been elucidated. Here, delayed oseltamivir treatment was used to mimic clinical settings on lethal influenza A (H1N1) pdm09 virus (pH1N1) infection mice model. We revealed that delayed oseltamivir plus sirolimus treatment protects mice against lethal pH1N1 infection by attenuating severe lung damage. Mechanistically, the combined treatment reduced viral titer and pH1N1-induced mTOR activation. Subsequently, it suppressed the NOD-like receptor family pyrin domain containing 3 (NLRP3) inflammasome-mediated secretion of interleukin (IL)-1β and IL-18. It was noted that decreased NLRP3 inflammasome activation was associated with inhibited nuclear factor (NF)-κB activation, reduced reactive oxygen species production and increased autophagy. Additionally, the combined treatment reduced the expression of other proinflammatory cytokines and chemokines, and decreased inflammatory cell infiltration in lung tissue and bronchioalveolar lavage fluid. Consistently, it inhibited the mTOR-NF-κB-NLRP3 inflammasome-IL-1β axis in a lung epithelial cell line. These results demonstrated that combined treatment with sirolimus and oseltamivir attenuates pH1N1-induced severe lung injury, which is correlated with suppressed mTOR-NLRP3-IL-1β axis and reduced viral titer. Therefore, treatment with sirolimus as an adjuvant along with oseltamivir may be a promising immunomodulatory strategy for managing severe influenza.

## Author summary

The severity and lethality of influenza A virus infection are frequently aggravated by virus-induced tissue destruction and overwhelming immune responses. Combined therapy with antiviral medications and immunomodulators, which not only inhibit viral replication, but also reduce the damaging consequences of host immune responses, will be beneficial in the treatment of severe influenza. In the present study, we revealed that pH1N1-induced activation of mTOR promotes lung immunopathological injury, which is correlated with upregulated NF-κB activity and increased reactive oxygen species production. Subsequently, it induces NLRP3 inflammasome activation and the secretion of IL-1β and IL-18. Combined treatment with oseltamivir and the mTOR inhibitor sirolimus (as an adjuvant) not only blocks viral replication, but also suppresses mTOR-NLRP3-IL-1β axis-mediated immune damage, thus protecting mice against lethal pH1N1 infection. Our findings provide the theoretical and experimental basis for the clinical investigation of sirolimus as an adjunct treatment for severe influenza.

## Introduction

Influenza A virus (IAV) infection represents a leading threat to global public health. New estimates have indicated that up to approximately 645,000 influenza-associated respiratory deaths occur annually [1]. Our previous clinical data showed that critically ill patients infected with influenza A (H1N1) pdm09 virus (pH1N1) is usually accompanied by acute lung injury (ALI) and acute respiratory distress syndrome (ARDS), which is characterized by sudden onset of respiratory failure, refractory hypoxemia, and noncardiogenic pulmonary edema, and pathologically by necrosis of bronchiolar walls, diffuse alveolar injury, and substantial inflammatory cell infiltration [2]. Our experimental and clinical studies on severe influenza infection have indicated that virus-induced tissue destruction and dysregulated systemic inflammation are associated with the severity and progression of the disease [2–7]. Combined therapy with antiviral medications and immunomodulators, which not only inhibit viral replication, but also reduce the damaging consequences of host immune responses, has been believed to be beneficial in the treatment for severe influenza pneumonia [8–10].

Rapamycin (sirolimus) is an inhibitor of mammalian target of rapamycin (mTOR). It not only blocks host pathways needed for viral replication [11–13], but also modulates the immune responses during infection [14–16]. Consequently, it may be a promising drug for treating influenza. It was reported that sirolimus combined with oseltamivir and corticosteroid treatment decreases viral titer and improves respiratory function in patients with severe H1N1 virus-induced pneumonia [17]. Furthermore, sirolimus contributes to delay the onset of morbidity in PR8 virus-infected mice [18]. Moreover, everolimus, a derivative of sirolimus, significantly reduces viral titer, lung weight, and hemorrhage score in H5N1 virus-infected mice [19]. However, the mechanisms of action of sirolimus plus oseltamivir in severe pneumonia induced by influenza infection have not been elucidated.

mTOR is a serine/threonine kinase that plays pivotal roles in cell survival, proliferation and metabolism [20]. It is composed of two different protein complexes: mTORC1, which directly phosphorylates S6 kinase (S6K), 4E-binding protein 1 (4EBP1), and subsequently activates the downstream target ribosomal protein S6 (S6RP); and mTORC2, which phosphorylates AKT [20]. It was reported that influenza virus HA and M2 protein promotes mTORC1 activation, and viral protein expression is mainly relied on mTORC1, but not mTORC2 [11]. The activation and functions of mTORC2 are not as well characterized as mTORC1. Previous studies suggested that mTORC2 may contribute to the regulation of apoptosis by the viral NS1 protein during infection [11, 21]. Aberrant mTOR activation plays critical roles in the pathogenesis of proinflammatory diseases including lipopolysaccharide induced-ALI, sepsis, atherosclerosis and neurodegenerative diseases, which make mTOR an important therapeutic target for these diseases [14]. Whether mTOR activation by influenza virus contributes to pathogenesis has not yet been fully elucidated.

NOD-like receptor family pyrin domain containing 3 (NLRP3) inflammasome is a multiprotein complex consisting of NLRP3, apoptosis-associated speck-like protein with CARD domain (ASC), and pro-caspase-1. It is a critical cytoplasmic pattern recognition receptor. Once NLRP3 inflammasome is activated, pro-caspase-1 undergoes autoactivation and cleavage to produce an enzymatically mature caspase-1, which further cleaves pro-IL-1β and pro-IL-18 into mature IL-1β and IL-18, respectively [22]. The NLRP3 inflammasome plays protective roles by initiating innate immune responses and promoting elimination of virus [23, 24]. Genetic deficiency of NLRP3, ASC, or caspase-1 in mice results in low concentrations of IL-1β and IL-18 in the bronchoalveolar lavage fluid (BALF) and serum, reduces the infiltration of leukocytes into the lungs, and increases viral titer [23–25]. However, recent studies suggest that excessive NLRP3 inflammasome activity can contribute to IAV-induced lung injury [26]. The use of a specific NLRP3 inhibitor has been noted to significantly protect mice from lethal influenza by reducing the BALF levels of proinflammatory cytokines, such as IL-1β and IL-18, and decreasing the recruitment of inflammatory cells into the lungs [27–29].

mTOR signaling has been reported to modulate the activity of NLRP3 inflammasome in different *in vivo* and *in vitro* models. Couchie et al. reported that the mTORC1/p-S6K signaling axis plays a key role in thioredoxin-80-induced inflammasome activity [30]. Furthermore, it has been shown that NLRP3 inflammasome-induced ischemia reperfusion injury can be averted by mTORC1 inhibition [31]. Additionally, mTORC1 can activate NLRP3 inflammasome in macrophages, whereas sirolimus sufficiently restricts NLRP3 inflammasome *via* mTORC1 inhibition [32]. Sirolimus can also induce the degradation of pro-IL-1β, and prevent maturation and secretion of cytokines by inhibiting NLRP3 inflammasome activity [33]. However, the effects of sirolimus on severe influenza virus-induced NLRP3 inflammasome activation have not yet been fully researched.

In the present study, we investigated the effects and mechanisms of delayed oseltamivir plus sirolimus on lethal influenza infection in mice. Our data showed that the combined treatment remarkably reduced ALI and systemic inflammation. Sirolimus may be a promising adjunctive therapy for severe viral pneumonia.

## Results

### Delayed oseltamivir plus sirolimus treatment attenuated pH1N1-induced mortality

**Fig 1:**
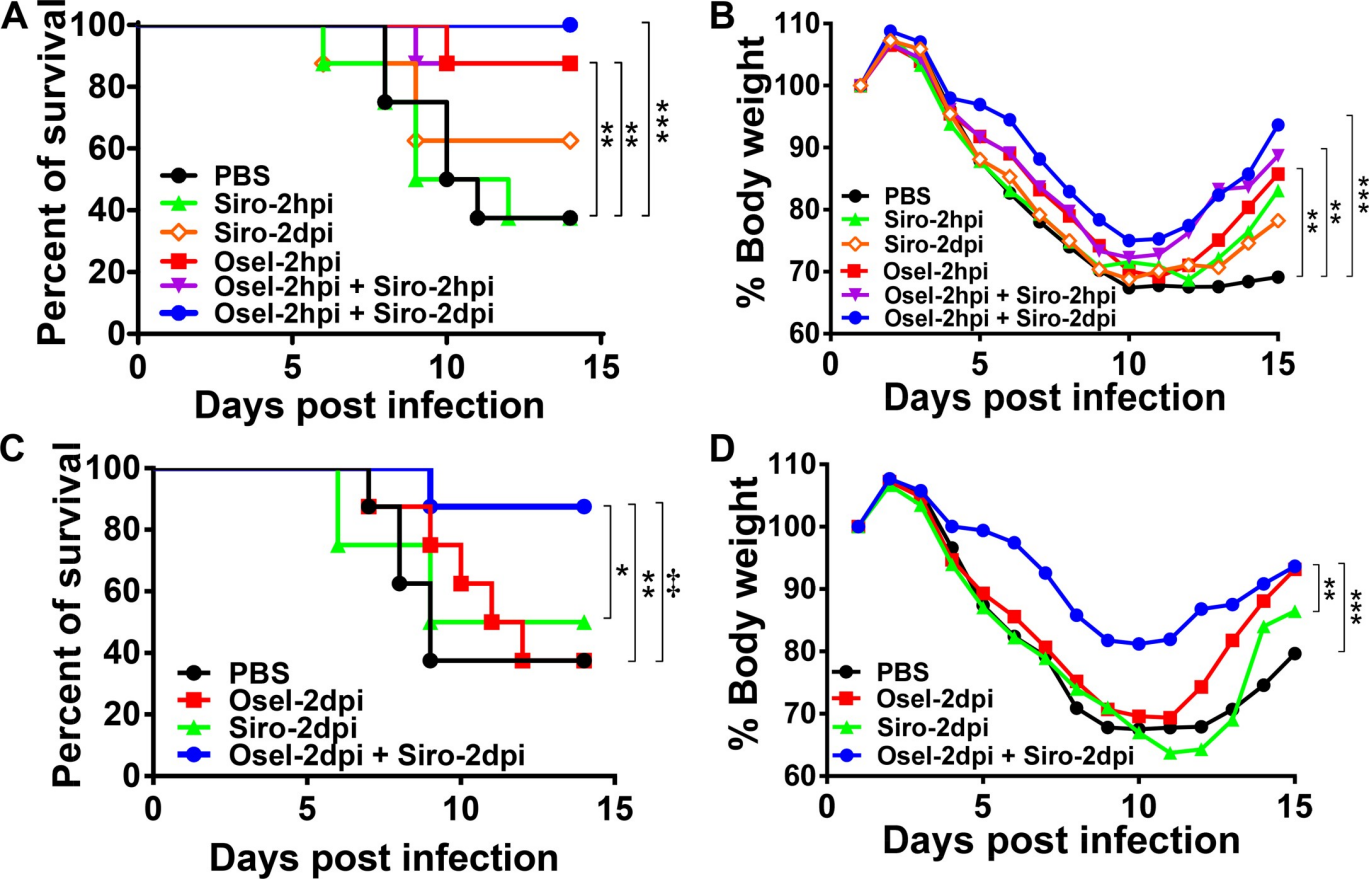
Delayed oseltamivir plus sirolimus treatment still attenuated pH1N1 virus-induced mortality. BALB/c mice were infected with pH1N1. Survival rate (A) and weight change ratio (B) following treatment with PBS, sirolimus (2 hpi), sirolimus (2 dpi), oseltamivir (2 hpi), oseltamivir (2 hpi) + sirolimus (2 hpi), or oseltamivir (2 hpi) + sirolimus (2 dpi). Survival rate (C) and weight change ratio (D) after treatment with PBS, oseltamivir (2 dpi), sirolimus (2 dpi), or oseltamivir (2 dpi) + sirolimus (2 dpi). Data are representative of two independent experiments (n = 8 for each group). In Fig 1A and 1B, ** and *** represent *p <* 0.01 and 0.001, respectively, when compared to the PBS-treated group. In Fig 1C, ** represent *p* < 0.01 when oseltamivir (2 dpi) + sirolimus (2 dpi) group compared to the oseltamivir group; ‡ represent *p* < 0.01 when oseltamivir (2 dpi) + sirolimus (2 dpi) group compared to the PBS group.

To explore the therapeutic potential of oseltamivir plus sirolimus, a mouse model of severe pH1N1 infection was established as described in our previous report [34]. As shown in Fig 1A, the mean survival time of mice in the PBS-treated control group was 11.1 ± 2.6 days, with the survival rate of only 37.5%. Sirolimus monotherapy did not result in a protective effect at 2 hours post infection (hpi); however, a slight protective effect was observed at 2 days post infection (dpi). Furthermore, oseltamivir monotherapy at 2 hpi, and combined therapy with oseltamivir at 2 hpi plus sirolimus at 2 hpi or 2 dpi resulted in a significant increase in survival rate. Additionally, body weight loss was less in mice in these groups compared to the PBS-control group at 14 dpi (Fig 1A and 1B).

Unfortunately, patients are usually not administered antiviral medications until 2–4 dpi after the onset of symptoms. Consequently, severe influenza-induced high mortality and morbidity are partially due to the delayed initiation of antiviral therapy. To simulate the clinical situation and real-life scenario, delayed oseltamivir and/or sirolimus treatment at 2 dpi were provided in subsequent experiments. Not surprisingly, delayed oseltamivir or sirolimus monotherapy did not confer any survival rate benefit. However, delayed therapy with both drugs resulted in a significantly higher survival rate than oseltamivir alone (87.5% *vs* 37.5%, *P* = 0.005) or sirolimus alone (87.5% *vs* 50%, *P* = 0.018) (Fig 1C). Consistent with the survival rate data, body weight loss was less with delayed combined therapy at 14 dpi (Fig 1D). These results demonstrate that combined therapy could provide significant survival benefits against lethal pH1N1 infection even if the treatment is delayed for 2 days.

### Delayed oseltamivir plus sirolimus treatment alleviated pH1N1-induced severe lung damage and decreased viral titer

**Fig 2:**
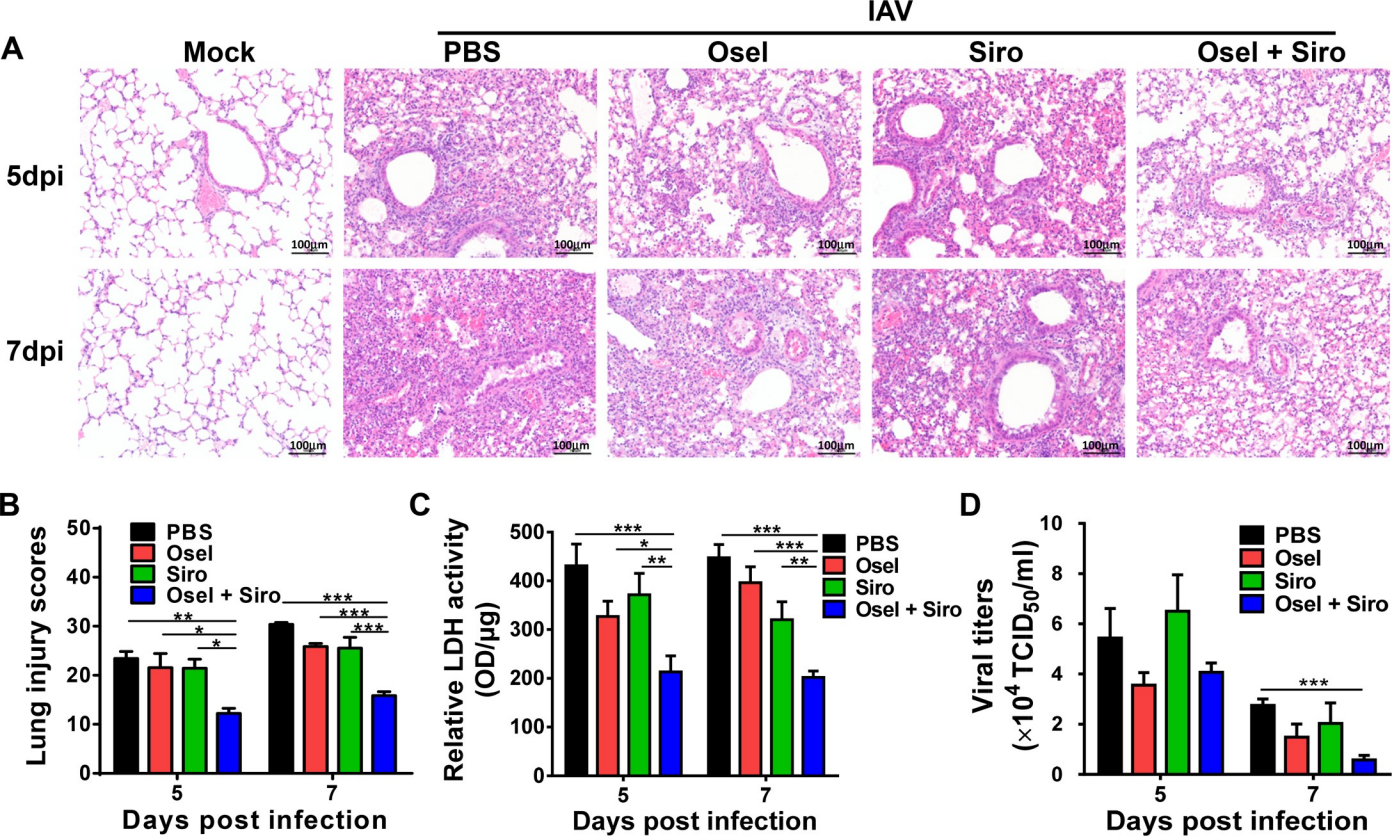
Delayed oseltamivir plus sirolimus treatment relieved pH1N1-induced severe lung damage. (A) Lung tissue injury was assessed by hematoxylin and eosin staining at 5 and 7 dpi. Scale bar = 100 μm, original magnification: ×200. (B) Semiquantitative histological scoring of lung injury. This experiment was done in a blinded manner. (C) Relative LDH activity in the bronchioalveolar lavage fluid of the infected mice following different treatments at the indicated dpi. (D) Viral titer in the lung homogenates of the infected mice following different treatments at the indicated dpi. Data are representative of two independent experiments and presented as mean ± SEM (n = 5 for each group). *, **, and *** represent *p* < 0.05, 0.01, and 0.001, respectively.

Excessive viral load and immune pathogenesis are important causes of severe lung injury [35–37]. To investigate the protective mechanisms of the combined therapy, pathological damage and viral titer in the lung were assessed. As shown in Fig 2A, PBS-treated pH1N1-infected lungs exhibited extensive lung damage, including desquamation of bronchiolar epithelial cells, diffused alveolar damage, and interstitial inflammatory infiltration at 5 dpi; moreover, lung damage at 7 dpi was more severe. Delayed oseltamivir or sirolimus monotherapy only slightly alleviated pathological damage, whereas delayed combined therapy significantly ameliorated lung pathological injury (Fig 2A and 2B). In addition, the activity of lactate dehydrogenase (LDH) in the BALF, which is representative of pathological damage to the lungs, was dramatically decreased in the delayed combined treatment group compared to the mock or monotherapy group (Fig 2C). Surprisingly, at 7 dpi, viral titer was also dramatically lower in the mice that underwent delayed combined treatment than in the PBS-treated mice (Fig 2D). These results suggest that delayed adjuvant sirolimus plus oseltamivir treatment synergistically alleviates lung immunopathological injury and decreases viral titer.

### Sirolimus significantly inhibited pH1N1-induced mTOR pathway activation *in vivo*

Previous studies have proved that mTOR activation plays important roles in diverse animal models of ALI [38–40]. Therefore, we investigated whether sirolimus alleviates ALI by suppressing pH1N1-induced mTOR pathway activation. The activation of mTOR and related molecules was assayed by western blotting and immunohistochemical analyses. The expression of phosphorylated (p)-mTOR and downstream p-S6K and p-S6RP were significantly increased in the lung tissues of pH1N1-infected mice (S1A Fig). Histological analysis further revealed the activation of the mTOR signaling pathway, as indicated by the expression of p-mTOR and p-S6RP, which were predominant in both bronchiolar and alveolar epithelial cells after infection with pH1N1 (S1B and S1C Fig).

**Fig 3:**
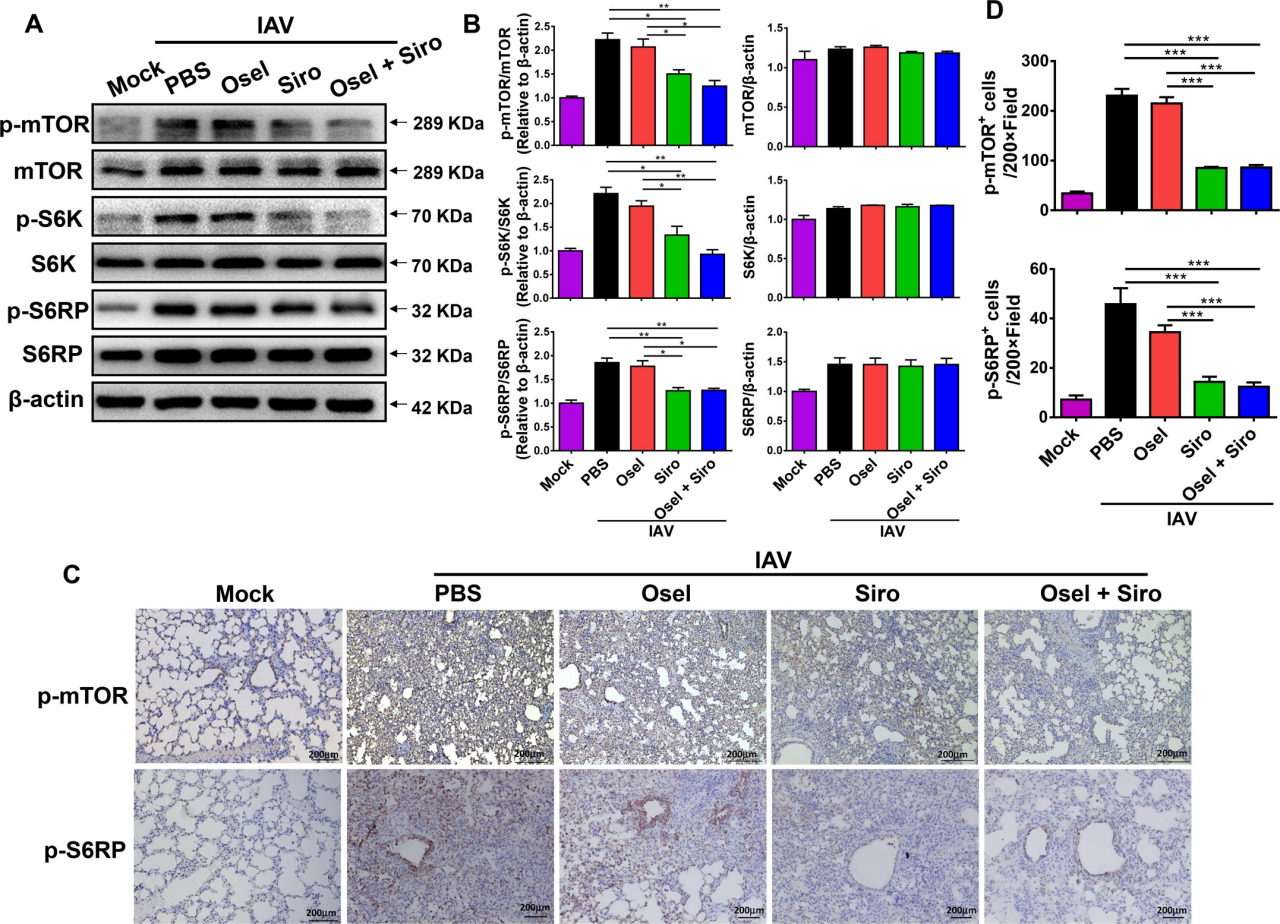
Sirolimus significantly inhibited pH1N1 virus-induced mTOR pathway activation *in vivo*. (A) Western blot analysis of p-mTOR, mTOR, p-S6K, S6K, p-S6RP, and S6RP expression in the lungs of different treatment groups at 5 dpi. (B) Expression of p-mTOR relative to mTOR, p-S6K relative to S6K, p-S6RP relative to S6RP, and the mTOR, S6K, and S6RP relative to β-actin in the different treatment groups. (C) Representative immunohistochemical images of p-mTOR and p-S6RP expression in the lungs of mice in the different treatment groups at 5 dpi. Scale bar = 200 μm, original magnification: ×100. (D) Quantitative analysis of p-mTOR- and p-S6RP-positive cells in the lungs of infected mice after the different treatments. Data are representative of two independent experiments and presented as mean ± SEM (n = 3 for each group). *, **, and *** represent *p* < 0.05, 0.01, and 0.001, respectively.

Next, we tested the ability of sirolimus to inhibit mTOR activation *in vivo*. The results showed that mTOR activation was lower in mice treated with sirolimus only or oseltamivir plus sirolimus than it was in the control mice or mice treated with oseltamivir only (Fig 3A and 3B). Moreover, immunohistochemical analysis of p-mTOR and p-S6RP further revealed that mTOR activity was markedly decreased in both alveolar and bronchiolar epithelial cells after treatment with sirolimus (Fig 3C and 3D). The results suggest that the mTOR pathway is activated during pH1N1 infection. Additionally, the pathway might mediate virus-induced lung injury; however, delayed treatment with sirolimus alleviates immunopathological lung injury *via* inhibition of mTOR activation.

### Improved ALI by delayed oseltamivir plus sirolimus treatment was correlated with suppressed NLRP3 inflammasome-mediated secretion of IL-1β and IL-18

**Fig 4:**
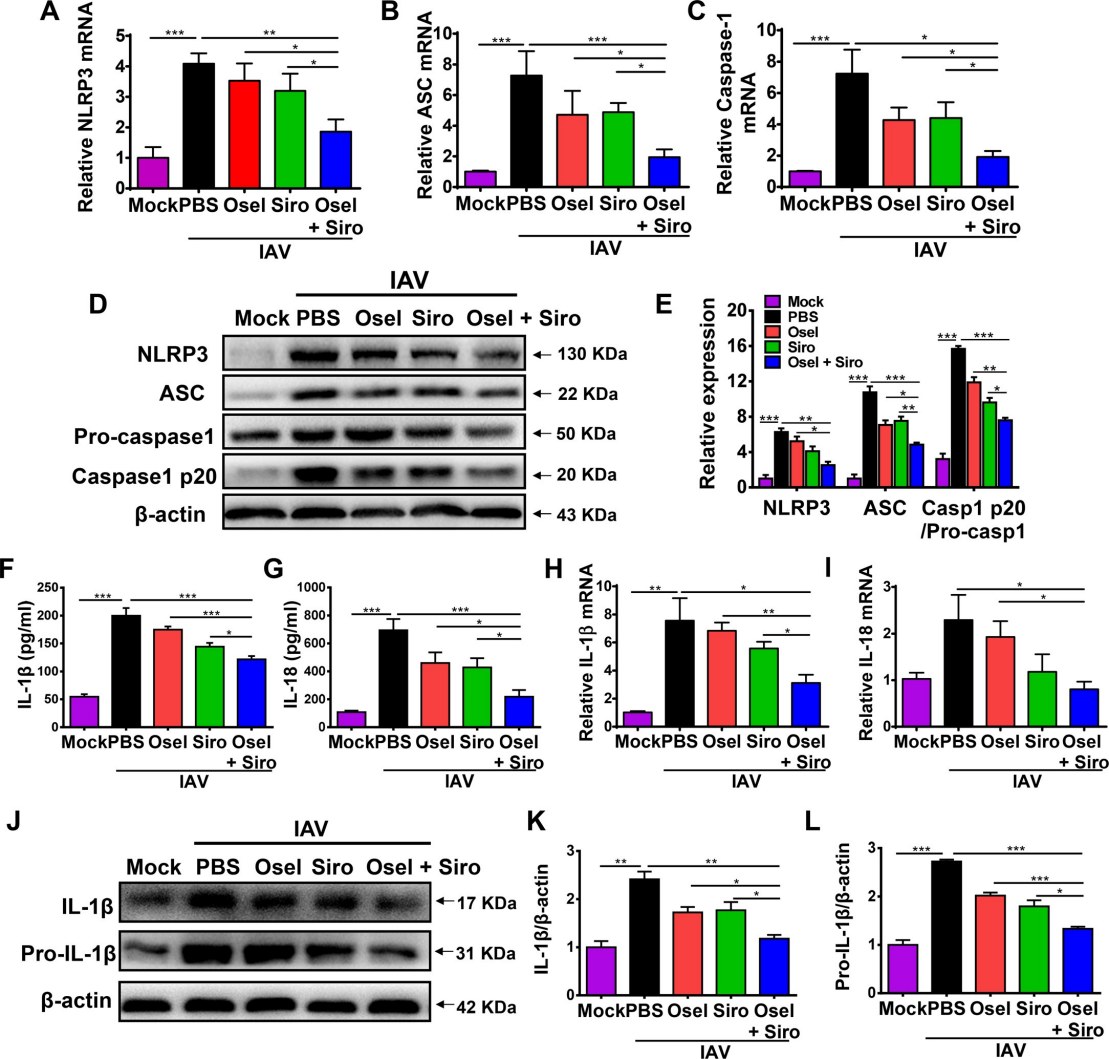
Improved ALI by delayed oseltamivir plus sirolimus treatment was correlated with suppressed NLRP3 inflammasome-mediated secretion of IL-1β and IL-18. Real-time PCR analysis of the NLRP3 inflammasome components, NLRP3 (A), ASC (B), and caspase-1 (C) in the lungs of infected mice in different treatment groups at 5 dpi. (D) Western blot analysis of NLRP3 inflammasome components in the lungs of infected mice in different treatment groups at 5 dpi. (E) Expression of NLRP3 and ASC relative to β-actin, and caspase 1 p20 relative to pro-caspase1. Concentrations of IL-1β (F) and IL-18 (G) in the BALF of infected mice in different treatment groups at 5 dpi. Real-time PCR analysis of IL-1β (H) and IL-18 (I) levels in the lungs of infected mice in different treatment groups at 5 dpi. (J) Western blot analysis of IL-1β and pro-IL-1β expression in the lungs of infected mice in different treatment groups at 5 dpi. Expression of IL-1β (K) and pro-IL-1β (L) relative to β-actin. Data are representative of two independent experiments and presented as mean ± SEM (n = 5 for each group). *, **, and *** represent *p* < 0.05, 0.01, and 0.001, respectively.

Excessive NLRP3 inflammasome activation contributes to aberrant inflammatory responses and causes severe ALI after influenza infection [26–29]. The NLRP3 inflammasome is a major downstream target of the mTOR signaling pathway [31, 32]. We next investigated whether the reduced immunopathological injury induced by sirolimus was associated with inhibition of NLRP3 inflammasome activation. As shown in Fig 4A–4E, delayed oseltamivir plus sirolimus treatment significantly decreased both mRNA and protein levels of NLRP3, ASC, and caspase-1 when compared with oseltamivir or sirolimus monotherapy (Fig 4A–4E). Once NLRP3 inflammasome is activated, pro-caspase-1 undergoes autocleavage to produce active caspase-1 (p20 subunit). The delayed combined treatment also significantly decreased the amount of active caspase-1 p20 in the lung tissue (Fig 4D and 4E).

Active caspase-1 can in turn cleave pro-IL-1β and pro-IL-18 into their mature forms. Influenza virus infection can dramatically induce the transcription and translation of pro-IL-1β and pro-IL-18 in the lung. The concentrations of IL-1β and IL-18 in the BALF at 5 dpi were markedly decreased by the combined treatment (Fig 4F and 4G). Additionally, lower IL-1β/IL-18 mRNA and IL-1β protein levels were also observed in the delayed combined treatment group than in the monotherapy group (Fig 4H–4L). These data suggest that the delayed combined therapy alleviates lung immunopathological damage, which is correlated with reduced NLRP3 inflammasome-mediated secretion of IL-1β and IL-18.

### Suppressed NLRP3 inflammasome activity was associated with inhibited nuclear factor (NF)-κB activation, reduced reactive oxygen species (ROS) production and elevated autophagy

**Fig 5:**
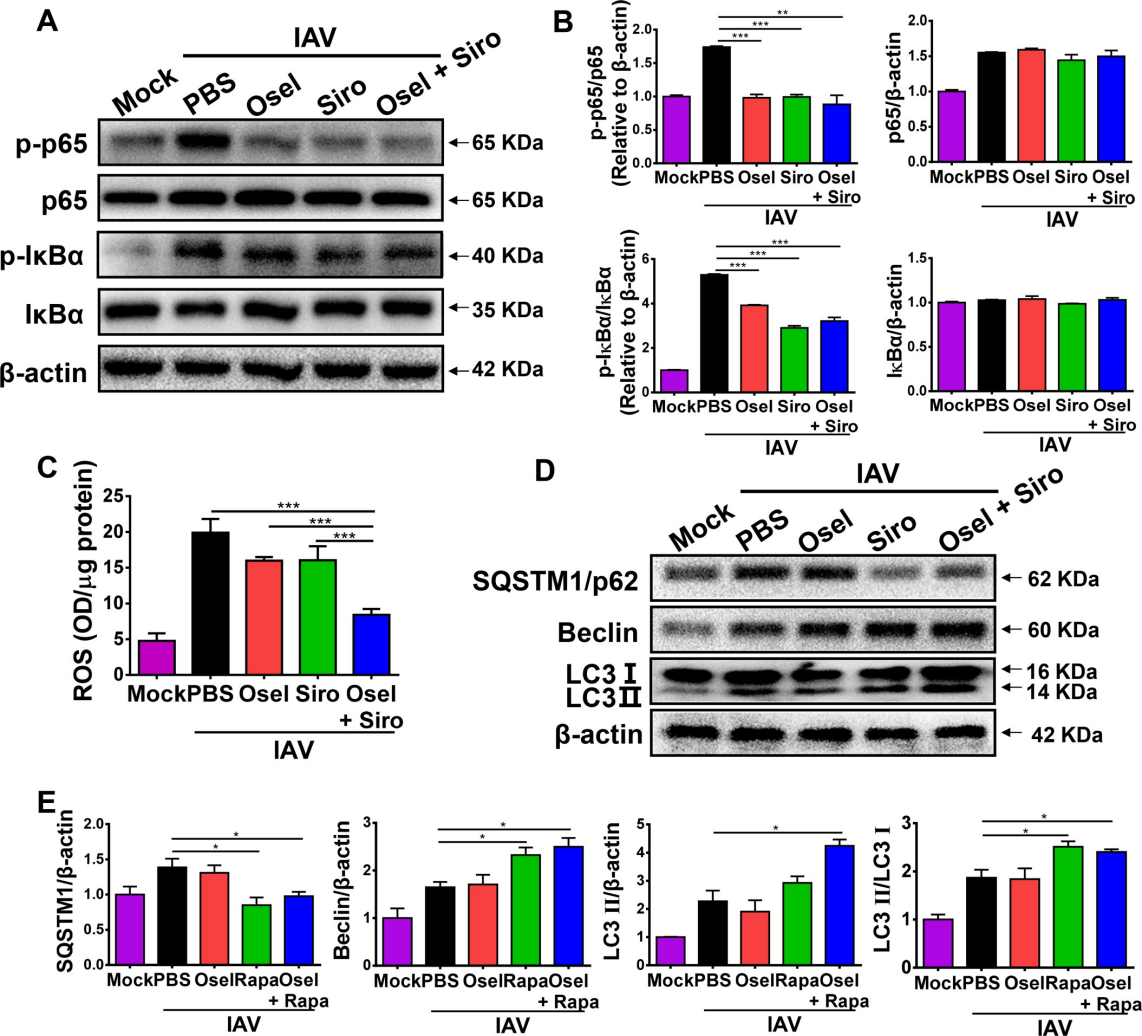
Suppressed NLRP3 inflammasome activity was associated with inhibited NF-κB activation, reduced ROS production, and increased autophagy. (A) Western blot analysis of p-p65, p65, p-IκB-α, and IκB-α expression in the lungs of infected mice in different treatment groups at 5 dpi. (B) Expression of p-p65 relative to p65, p-IκB-α relative to IκB-α, and p65 or IκB-α relative to β-actin. (C) ROS content in the lung homogenates of infected mice in different treatment groups at 5 dpi. (D) Western blot analysis of SQSTM1/p62, Beclin and LC3 I/II expression in the lungs of infected mice in different treatment groups at 5 dpi. (E) Expression of SQSTM1/p62, Beclin, and LC3 II relative to β-actin, and LC3 II relative to LC3 I in the different treatment groups. Data are representative of two independent experiments and presented as mean ± SEM (n = 5 for each group). *, **, and *** represent *p* < 0.05, 0.01, and 0.001, respectively.

The potential mechanisms underlying reduced NLRP3 inflammasome activity by the delayed combined therapy were investigated. NF-κB and ROS are well-recognized factors that cooperatively mediate the activation of NLRP3 inflammasome [41–43]. Influenza virus induced NF-κB activation through phosphorylation of p65 and IκBα. Treatment with sirolimus and/or oseltamivir effectively decreased the levels of p-p65 and p-IκBα in the lungs of the virus-infected mice (Fig 5A and 5B). Moreover, the delayed combined therapy, but not monotherapy, significantly reduced ROS production in the lung homogenates (Fig 5C). Sirolimus can also induce autophagy to inhibit the NLRP3 inflammasome activation [44]. The autophagy-related hallmarks were also measured in pH1N1-infected mice. As shown in Fig 5D and 5E, delayed sirolimus or sirolimus plus oseltamivir treatment could dramatically induce autophagic flux, including decreased expression of autophagy substrate SQSTM1/p62, as well as increased expression of autophagosomes protein Beclin and LC3-II in the lung tissue at 5 dpi. These data suggest that delayed oseltamivir plus sirolimus treatment reduces NLRP3 inflammasome activity, which is correlated with downregulated NF-κB activity and ROS production, and induced autophagy after pH1N1 infection.

### Delayed oseltamivir plus sirolimus treatment decreased the expression of various proinflammatory cytokines and chemokines

**Fig 6:**
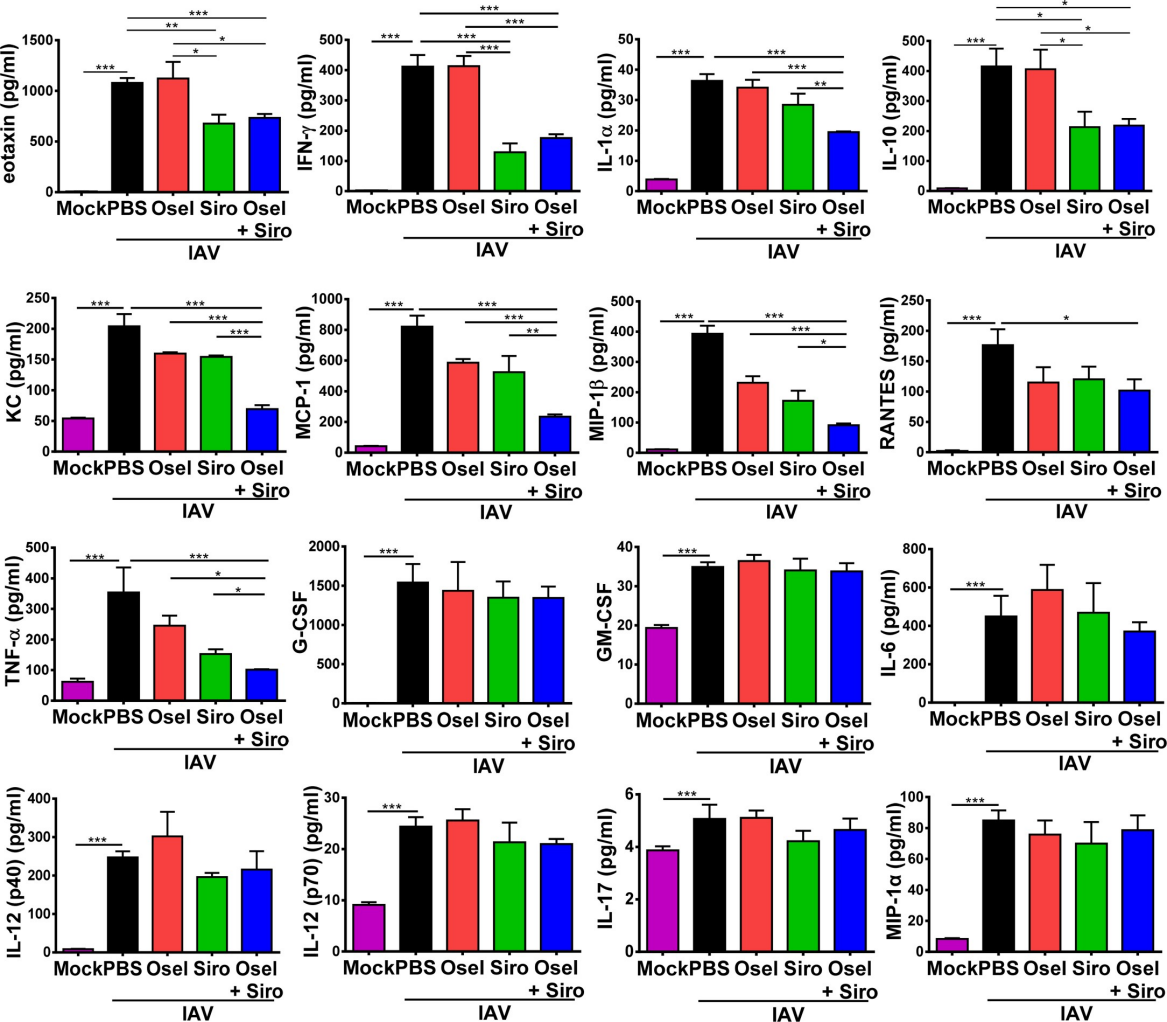
Delayed oseltamivir plus sirolimus treatment decreased the levels of various proinflammatory cytokines and chemokines in the BALF. The concentrations of eotaxin, IFN-γ, IL-1α, IL-10, KC, MCP-1, MIP-1β, RANTES, TNF-α, G-CSF, GM-CSF, IL-6, IL-12 (p40), IL-12 (p70), IL-17 and MIP-1α in the BALF of infected mice in different treatment groups at 7 dpi. Data are representative of two independent experiments and presented as mean ± SEM (n = 5 for each group). *, **, and *** represent *p* < 0.05, 0.01, and 0.001, respectively.

We have reported that cytokine storm contributes to pH1N1-induced lung immunopathological damage [4, 5]. To further elucidate whether the delayed oseltamivir plus sirolimus treatment can repress the cytokine storm after pH1N1 infection, the concentrations of multiple cytokines/chemokines in BALF were measured at 7 dpi using Bio-Plex. The delayed combined therapy significantly decreased the concentrations of eotaxin, IFN-γ, IL-1α, IL-10, KC, MCP-1, MIP-1β, RANTES and TNF-α compared with untreated control or monotherapy group; however, the levels of G-CSF, GM-CSF, IL-6, IL-12 (p40), IL-12 (p70), IL-17 and MIP-1α were not noticeably affected (Fig 6).

### Delayed oseltamivir plus sirolimus treatment reduced inflammatory cell infiltration

**Fig 7:**
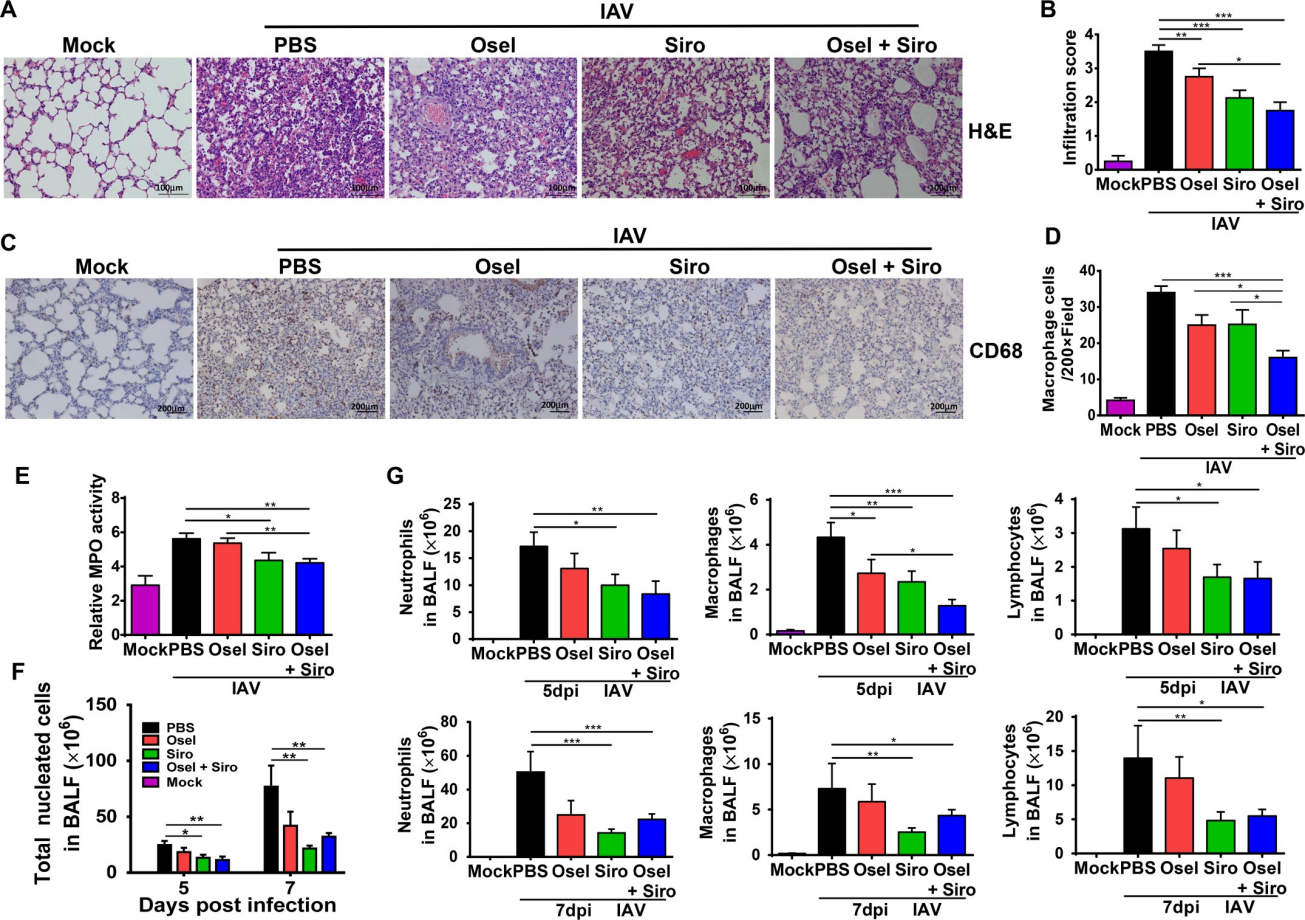
Delayed oseltamivir plus sirolimus treatment reduced inflammatory cell infiltration. (A) Histopathological examination of the inflammatory cell infiltration into the lungs of infected mice in different treatment groups at 5 dpi. Scale bar = 100 μm. (B) Lung inflammatory cell infiltration scores of infected mice in different treatment groups. (C) Representative immunohistochemical images of CD68 expression in the lungs of the infected mice in different treatment groups at 5 dpi. Scale bar = 200 μm, original magnification: ×100. (D) Quantitative analysis of CD68-positive cells in the lungs of infected mice in different treatment groups. (E) Relative MPO activity in the lung homogenates of infected mice in different treatment groups at 5 dpi. (F) Total cell counts and (G) the numbers of neutrophils, macrophages, and lymphocytes in BALF samples from different treatment groups at 5 and 7 dpi. Data are representative of two independent experiments and presented as mean ± SEM (n = 5 per group). *, **, and *** represent *p* < 0.05, 0.01, and 0.001, respectively.

Abrupt increases in the levels of cytokines and chemokines may contribute to cell recruitment. We then measured the effect of combination treatment on inflammatory cell infiltration after pH1N1 infection. As shown in Fig 7A and 7B, pH1N1 infection caused a large amount of leukocytes infiltration, whereas delayed sirolimus or oseltamivir plus sirolimus treatment significantly reduced inflammatory cell infiltration into the lungs, which was evidenced by low infiltration scores (Fig 7A and 7B). Particularly, the delayed combined therapy significantly decreased macrophage infiltration into the lung tissue (Fig 7C and 7D). Myeloperoxidase (MPO) activity, which reflects the number of neutrophils in the lung, was also dramatically decreased after the treatment with sirolimus or oseltamivir plus sirolimus (Fig 7E).

The total number of cells and the numbers of different cell types in BALF were also examined. Delayed sirolimus or oseltamivir plus sirolimus treatment significantly reduced the total number of leukocytes, as well as the number of neutrophils, macrophages and lymphocytes at 5 and 7 dpi (Fig 7D and 7E). These data suggest that the delayed combined therapy can alleviate cytokine storm and inflammatory cell infiltration.

### Combined oseltamivir and rapamycin treatment inhibited viral spread and reduced mTOR-NF-κB-NLRP3 inflammasome-IL-1β signaling in lung epithelial cells *in vitro*

Airway epithelial cells are the primary targets of influenza virus. We had demonstrated that the viral titers were synergistically reduced in combined treatment group at 7 dpi *in vivo* (Fig 2D). To further confirm the impact of rapamycin on viral replication, mouse lung epithelial cells (MLE12) were treated with oseltamivir and/or rapamycin after pH1N1 infection *in vitro*. The copies of viral RNA genomes in the cell-free supernatant were measured in control, oseltamivir, rapamycin, and oseltamivir plus rapamycin treatment groups. As shown in S2 Fig, oseltamivir, as well as rapamycin, could significantly decrease the copies of viral RNA genomes in the cell-free supernatant. Moreover, the combined oseltamivir and rapamycin treatment further decreased the copies of viral RNA genomes at 24 hpi (S2 Fig). The results suggested that rapamycin could decrease viral replication through inhibiting mTOR-mediated initiation and enhancement of viral mRNAs translation *in vitro*.

**Fig 8:**
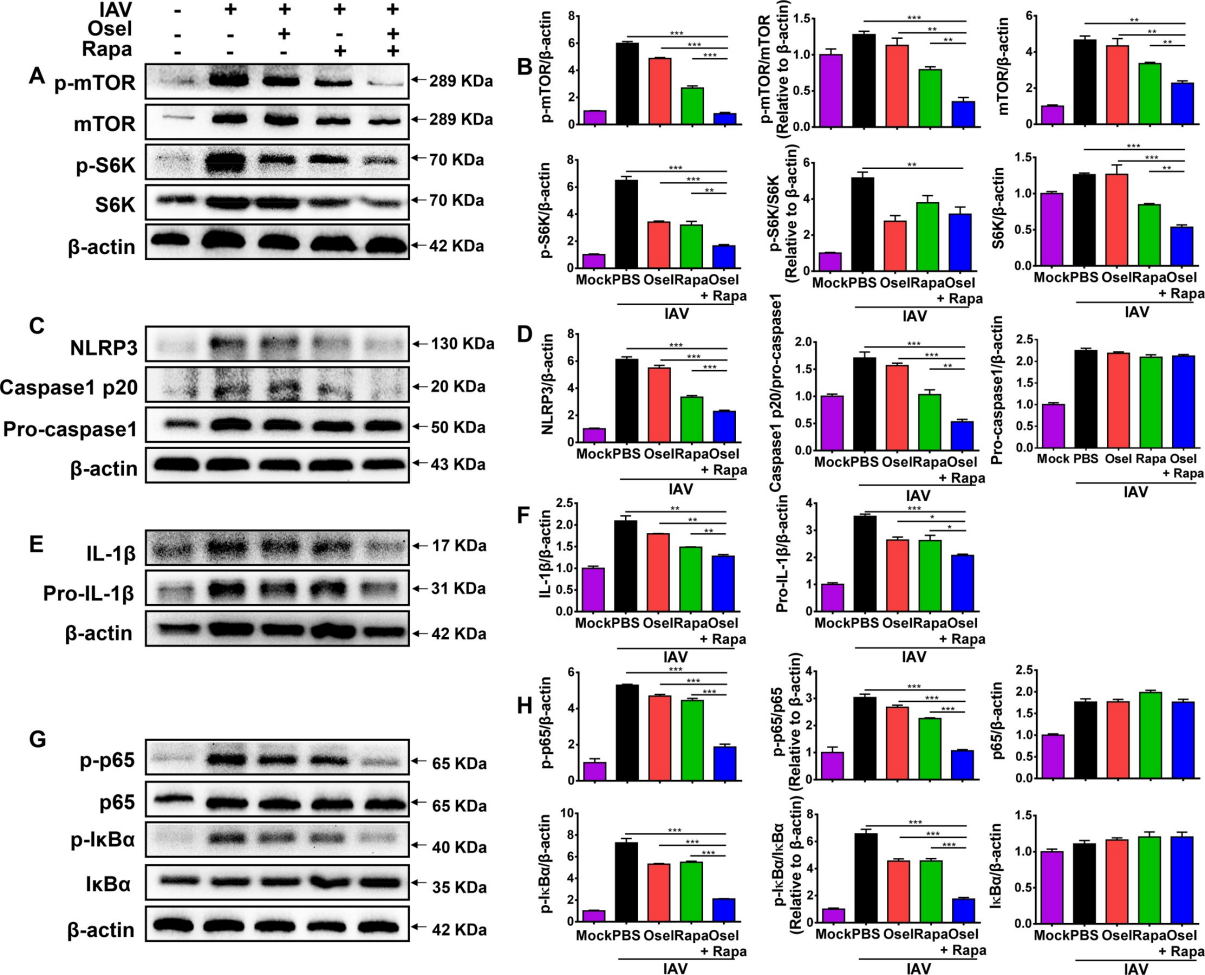
Combined oseltamivir plus sirolimus treatment reduced mTOR-NF-κB-NLRP3 inflammasome-IL-1β axis in lung epithelial cells. Infected MLE12 cells were harvested and analyzed for protein expression by western blotting. (A) Protein levels of p-mTOR, mTOR, p-S6K, and S6K. (B) Expression of p-mTOR relative to mTOR, p-S6K relative to S6K, and p-mTOR, mTOR, p-S6K, and S6K relative to β-actin. (C) Protein levels of NLRP3, caspase-1 p20, and pro-caspase-1. (D) Expression of NLRP3 and pro-caspase-1 relative to β-actin, and caspase-1 p20 relative to pro-caspase-1. (E) Protein levels of IL-1β and pro-IL-1β. (F) Expression of IL-1β and pro-IL-1β relative to β-actin. (G) Protein levels of p-p65, p65, p-IκB-α, and IκB-α. (H) Expression of p-p65 relative to p65, p-IκB-α relative to IκB-α, and p-p65, p65, p-IκB-α, and IκB-α relative to β-actin. Data are representative of two independent experiments and presented as mean ± SEM. *, **, and *** represent *p* < 0.05, 0.01, and 0.001, respectively.

In addition to hematopoietic cells, NLRP3 inflammasome activation also occurs in non-immune cells, such as primary bronchial epithelial cells, lung fibroblasts, and various epithelial cell lines infected with influenza virus [25, 35, 41, 45, 46]. To further confirm the effects of the combined treatment on the mTOR-NF-κB-NLRP3 inflammasome-IL-1β pathway, the activation of mTOR and related downstream molecules S6K were assayed by western blotting. As expected, the levels of p-mTOR and p-S6K were dramatically increased after pH1N1 infection in MLE12 cells, whereas oseltamivir and rapamycin reduced mTOR activity synergistically (Fig 8A and 8B). Meanwhile, H1N1 virus-induced NLRP3 inflammasome activation was synergistically repressed by the combined treatment. This was shown by the significantly low levels of NLRP3 and activated caspase-1 p20 (Fig 8C and 8D). Consistently, the combined treatment further reduced NLRP3 inflammasome-mediated maturation and secretion of IL-1β (Fig 8E and 8F). Mechanistically, oseltamivir plus rapamycin synergistically inhibited NF-κB signaling by significantly decreasing p-p65 and p-IκB expression in MLE12 cells (Fig 8G and 8H). Because rapamycin inhibits viral replication, and may further cause decreased immune responses. To rule out the effect of rapamycin induced virus titer depression on immune responses, the MLE12 cells were treated with oseltamivir and/or rapamycin without pH1N1 infection *in vitro*. As shown in S3 Fig, oseltamivir had no effect on the expression of NLRP3 and IL-1β, however, rapamycin or rapamycin plus oseltamivir significantly decreased the expression of NLRP3 and IL-1β in MLE12 cells. The results suggested that rapamycin could directly modulate the host immune responses. Furthermore, rapamycin induced autophagy-related hallmarks were also measured *in vitro* experiments. Consistent with the results of the *in vivo* studies, oseltamivir plus rapamycin treatment also significantly decreased the expression of autophagy substrate SQSTM1/p62 and increased the expression of autophagosomes protein Beclin or LC3-II in MLE12 cells at 24, 48 or 72 hpi (S4 Fig). All the data demonstrate that combined oseltamivir and rapamycin treatment reduces the NLRP3 inflammasome-mediated secretion of IL-1β in lung epithelial cells, which is correlated with inhibited mTOR-NF-κB signaling and induced autophagy.

## Discussion

Pandemic influenza infection causes substantial mortality. Oseltamivir is most effective against the infection only when it is administered within 48 h from the onset of symptoms. However, in clinical settings, patients usually can’t receive treatment until overt illness symptoms occur after infection, by which time viral load may already be high. Therefore, the high inoculum and delayed therapy used in the presently reported mouse model can better simulate the real clinical situation.

Host immune responses that are elicited by IAV infection play important roles in promoting viral clearance. However, aberrant or uncontrolled immune responses, such as cytokine storm and excessive cellular activation, are also important in the pathogenesis of influenza-induced ALI [35, 37, 47]. Previous reports have suggested that once the viral infection triggers a cytokine storm, proinflammatory cytokines and chemokines continue to induce immunopathological damage even if viral replication is suppressed [48]. All the results indicated that overwhelming immune responses and viral virulence were the main reasons for the severe disease or even death [2–7, 37, 49–53]. Adjuvant corticosteroid therapies are often used in the management of patients with ALI/ARDS, as they are hoped to reduce lung inflammation and improve clinical outcomes. However, clinical data of our study as well as results of other groups have demonstrated that adjuvant corticosteroid therapy may be harmful in patients with influenza pneumonia [54–59]. Novel drugs that attenuate the severity of the infection by reducing the inflammatory response might be promising agents for influenza treatment.

**Fig 9:**
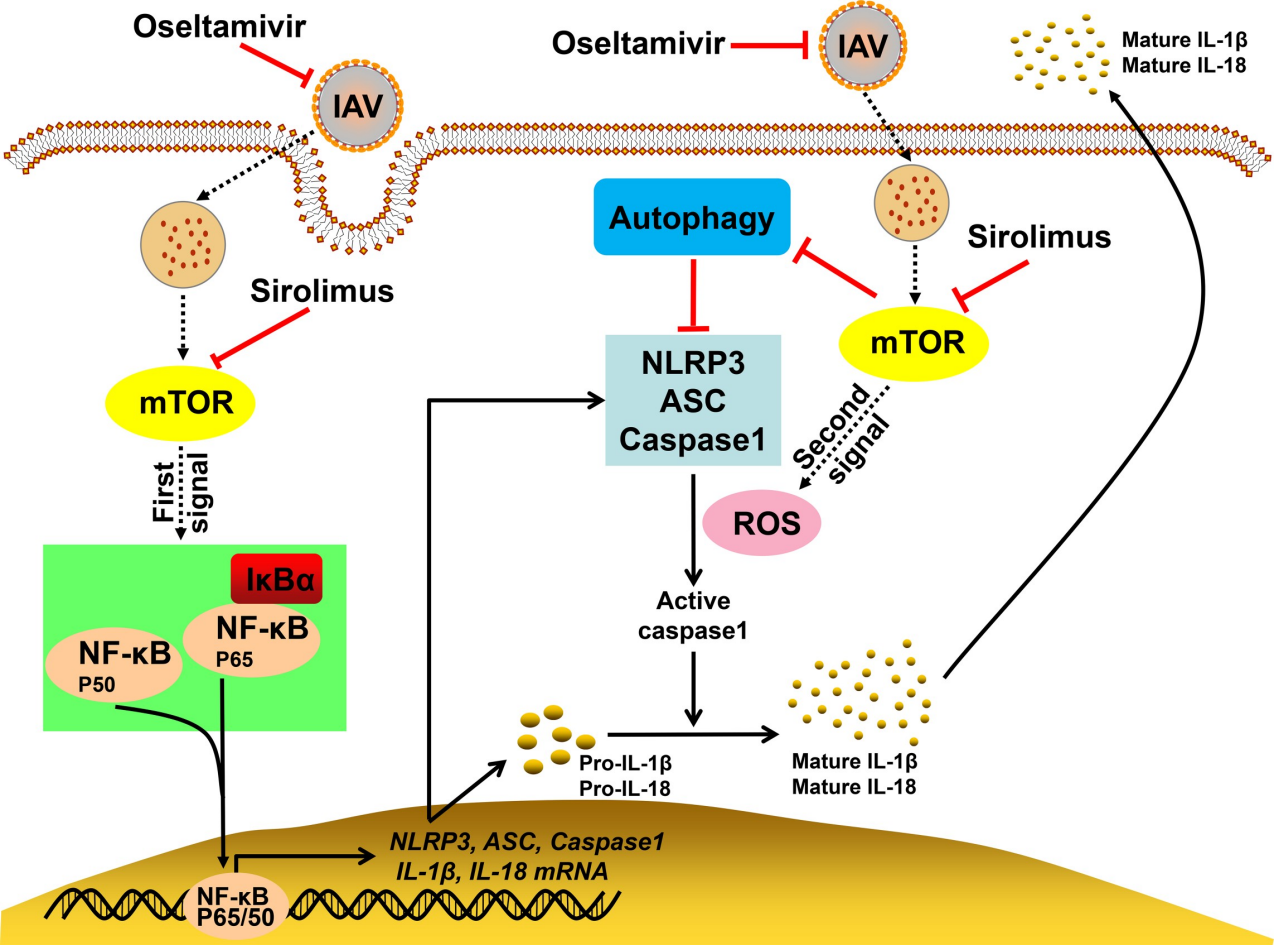
Graphic summary of the mechanisms underlying the effects of the combined therapy. After pH1N1 virus infection, mTOR signaling is activated, which promotes NF-κB activity and ROS production, and leads to the activation of NLRP3 inflammasome. Two signals are needed for the activation of NLRP3 inflammasome. Signal one is associated with the activation of the transcription factor NF-κB, which upregulates the transcription and expression of NLRP3, ASC, caspase-1, pro-IL-1β, and pro-IL-18. Signal two is mediated by pH1N1 virus-induced ROS production. The two signals cooperatively mediate the assembly of NLRP3 inflammasome and the cleavage of pro-caspase-1 into active caspase-1. The active caspase-1 can further cleave pro-IL-1β and pro-IL-18 into activated IL-1β and IL-18, respectively, and induce their secretion out of the cells. The bioactive IL-1β and IL-18 produced by NLRP3 inflammasome promotes pH1N1-induced lung injury. Sirolimus inhibits the activation of NF-κB by targeting mTOR. This decreases the expression of NLRP3 inflammasome components, pro-IL-1β and pro-IL-18. Furthermore, sirolimus induces autophagy and reduces ROS production to inhibit NLRP3 inflammasome assembly, which further decreases IL-1β and IL-18 release. Subsequently, there is a decline in the levels of various cytokines and chemokines, which reduces inflammatory cell infiltration into the lungs and BALF. As a result, combined therapy with oseltamivir and sirolimus inhibits viral replication and attenuates virus-induced severe immunopathological lung injury.

In the present study, we found that the delayed oseltamivir plus sirolimus treatment significantly improves survival rate and reduces inflammatory lung tissue damage during pH1N1 infection. We noted that sirolimus potently reduces pH1N1 virus-induced mTOR activation, and subsequently suppresses the NLRP3 inflammasome-mediated secretion of IL-1β and IL-18. The decreased activation of NLRP3 inflammasome by sirolimus was associated with inhibited NF-κB activation, reduced ROS production, and increased autophagy (Fig 9). Furthermore, the delayed combined treatment significantly reduced the BALF levels of various cytokines and chemokines. Additionally, inflammatory cell infiltration into the lungs and BALF was reduced after combination treatment. Consistent with the results of the *in vivo* studies, oseltamivir plus rapamycin reduced mTOR-NF-κB-NLRP3 inflammasome-IL-1β axis signaling in epithelial cells.

The mTOR inhibitor sirolimus is widely used in transplant medicine and oncology. It has pivotal roles in immune responses and cellular metabolism. The mTOR inhibition not only leads to the reduction of NF-κB-mediated inflammatory cytokine production [60], but also results in the potent suppression of CD4^+^ effector T cells and the promotion of Treg cells [61, 62]. It has been shown that organ transplant patients who received mTOR inhibitors exhibits decreased efficacy of H1N1 pandemic vaccination [63].

A recent report indicated that influenza virus induces metabolic phosphatidylinositide 3-kinase (PI3K)/mTOR changes; however, the PI3K/mTOR inhibitor BEZ235 restores PI3K/mTOR pathway homeostasis and significantly reduces viral titer to alleviate lethal influenza infection [64]. We found that rapamycin significantly decreased the copies of viral RNA genomes in the cell-free supernatant *in vitro*. Although sirolimus monotherapy could inhibit pH1N1-induced excessive immune responses (Figs 5, 6 and 7), which may affect host anti-viral capability, the viral titers were not increased at 5 or 7 dpi *in vivo*. These results suggested that sirolimus may have the ability to depress viral replication through inhibiting mTOR-mediated initiation and enhancement of viral mRNAs translation.

As shown in Fig 2, although the viral titers were reduced after oseltamivir monotherapy at 5 dpi and 7 dpi, the lung injury was not significantly improved in our animal experiments. Otherwise, although the viral titers in oseltamivir and sirolimus combined therapy group were only slightly lower compared with PBS treatment control group at 5 dpi, the combined therapy still showed significantly improved pathological damage and less body weight loss (Fig 2 and Fig 1D). Meanwhile, the combined therapy significantly decreased the levels of some proinflammatory cytokines and chemokines, as well as cell infiltration (Figs 6 and 7). The results suggested that the depression of excessive immune responses by sirolimus might be one of important mechanisms for the enhanced protection following infection. In addition, although sirolimus monotherapy partially inhibited the dysregulated immune responses (Figs 6 and 7), it only slightly improved the survival rate (Fig 1C). The results indicated that antiviral treatment using oseltamivir to depress viral titers and reduce virus-induced cell death is also necessary. Modulating dysregulated immune responses and inhibiting viral titers can ameliorate immunopathological injury of lung.

The assembly of the NLRP3 inflammasome results in the activation of caspase-1 and mediates the processing and release of IL-1β. Consequently, it plays central roles in inflammatory responses in diverse human diseases. However, NLRP3 inflammasome signaling must be tightly controlled to avoid inducing a hyperinflammatory state after lethal influenza infection [26–28, 41, 65]. The mTORC1 signaling axis plays key roles in regulating NLRP3 inflammasome through multiple mechanisms [30–33, 44]. Our data showed that sirolimus significantly suppressed the NLRP3 inflammasome-mediated secretion of IL-1β and IL-18. During IAV infection, two signals are required for NLRP3 inflammasome activation [42]. Firstly, NF-κB activation is needed to increase the expression of pro-IL-1β and pro-IL-18, as well as inflammasome components, NLRP3, ASC, and pro-caspase-1. Secondly, the NLRP3 inflammasome is activated by stimuli including potassium efflux, lysosomal maturation, and ROS production [43]. Several studies have shown that there is a positive crosstalk between mTOR and NF-κB/ROS during inflammation [44, 66–71]. Consistent with those data, our results showed that sirolimus significantly inhibited NF-κB and ROS by inhibiting mTOR. Furthermore, we found that combination treatment significantly increased autophagy, which may further reduce ROS and inhibit the NLRP3 inflammasome [44]. In conclusion, our results suggest that sirolimus suppresses NLRP3 inflammasome, which is correlated with inhibited NF-κB activation and ROS production, and induced autophagy after pH1N1 infection; however, it is not clear whether other inflammasomes, such as AIM2, are also inhibited.

Our experimental and clinical studies have revealed that dysregulated cytokine storm and excessive inflammatory cell infiltration are associated with disease severity [2–6]. In the present study, our results showed that the combined treatment significantly decreased the concentrations of cytokines and chemokines in BALF. Moreover, immune cell recruitment into the lungs and BALF was also reduced. Inhibition of immune cell infiltration and the expression of inflammatory mediators might have contributed to the beneficial effects of the combination treatment. It has been found that inflamed neutrophils contribute to aberrant pulmonary inflammation and ARDS during influenza infection [52, 72]. We found that the combined treatment significantly reduces neutrophil count in the BALF and lung tissue. It has been reported that inflamed macrophages contribute to secondary bacterial infections, which ultimately result in morbidity and mortality [73]. We also confirmed that the combined treatment significantly reduces the number of macrophages in the BALF and lung tissue. However, it was not clarified if oseltamivir plus sirolimus can prevent bacterial infections after IAV infection.

A few studies have shown that sirolimus increases disease severity after influenza infection. Alsuwaidi et al. reported that sirolimus treatment exacerbates respiratory function by increasing viral titer and worsening lung inflammation [74]. Huang et al. found that sirolimus treatment accelerates disease progression and abolishes the protective effect of oseltamivir treatment by suppressing antigen-specific T cell immunity and impairing virus clearance [75]. However, our results showed protective roles of sirolimus against severe infection when combined with oseltamivir. Possible explanations for this discrepancy include differences in the administration route, dosage, and administration or maintenance time of sirolimus. The prerequisite immune responses to virus infection may be completely suppressed by high dosage of sirolimus (3–5 μg/g) [74, 75], and low dosage of sirolimus (0.03 μg/g) may have no effect on the severe influenza infection-induced excessive immune response [75]. As an immunosuppressant drug, improper medication is not conducive to achieve the homeostasis of immune response, which not only inhibits excessive immune response, but also does not impede cellular and humoral responses to promote virus clearance. The dosage of sirolimus used in our study (initial dosage, 0.6 μg/g; maintenance dosage, 0.3 μg/g) was similar to those used in a clinical trial (2 mg/day) and previous studies in mice (0.6–1 μg/g) which indicated the protective roles of sirolimus [17, 18]. Moreover, using different virus strains and infective doses may lead to different results. Lethal dose of IAV A/PR/8/34 (H1N1) (10^8.1^ TCID_50_ or 1.25 × 10^4^ PFU) [74, 75], and sub-lethal dose (2.5 × 10^3^ PFU) of IAV A/PR/8/34 (H1N1) [75] were used. The virus strain and infective dose used in our study can better simulate the clinical situation. The detailed mechanisms as to why sirolimus can be either protective or deleterious in lung injury should be further investigated.

In conclusion, our findings show that delayed combined oseltamivir plus sirolimus therapy protects against lethal pH1N1 infection by inhibiting lung immunopathologic injury. The combined therapy inhibited viral replication, restored PI3K/mTOR pathway homeostasis, and repressed inappropriate aggressive immune responses. This indicates that sirolimus can be used as a novel treatment alongside existing antiviral therapies in severe influenza. Our findings provided the theoretical and experimental basis for its use in further clinical trials. Future studies investigating detailed medication time and dosage strategies will contribute to the development of sirolimus as an effective adjunct in the treatment of influenza infection.

## Materials and methods

### Mice, influenza virus and cells

Specific pathogen free female BALB/c mice, at 6–8 weeks old, were obtained from the Institute of Laboratory Animal Sciences, Beijing, China.

Influenza A (H1N1) pdm09 virus was amplified for 3 days at 37°C in the allantoic cavities of 10-day-old embryonated chicken eggs. Clarified allantoic fluid was aliquoted and immediately frozen at −80°C until use.

Madin-Darby canine kidney cells (MDCK) cells were obtained from American Type Culture Collection (ATCC). MDCK cells were maintained in Eagle’s minimal essential medium (EMEM) (Gibco, Life Technologies, NY) supplemented with 10% fetal bovine serum (FBS) (Gibco, Life Technologies, NY), 100 μg/ml streptomycin and 100 IU/ml penicillin (Gibco, Life Technologies, NY), and were cultured at 37°C in 5% CO_2_.

Mouse lung epithelial cells (MLE12) (ATCC) were maintained in Dulbecco's modified eagle medium (DMEM/F-12) (Gibco, Life Technologies, NY) supplemented with 10% fetal bovine serum (FBS), 100 μg/ml streptomycin and 100 IU/ml penicillin, and were cultured at 37°C in 5% CO_2_.

### Influenza virus infection

Mice were anesthetized and inoculated intranasally with 10^2^ 50% tissue culture infective doses (TCID_50_) of virus in a total volume of 50 μl. Control mice were administered with an equal volume of PBS. The TCID_50_ was determined in MDCK cells after serial dilution of the stock. After infection, mice were weighed and survival of mice was monitored for up to 14 days post infection or until death.

### Oseltamivir and rapamycin treatment

Oseltamivir (Roche, Basel, Switzerland) and sirolimus (Pfizer, NY) were administered by gavage once per day for consecutive 7 days at the indicated days post infection. The administered dosage for oseltamivir was 30 mg/kg. For the mice treated with sirolimus, a loading dose of 600 μg/kg was used on the first day of treatment to facilitate achievement of steady-state blood concentrations, a maintain dose of 300 μg/kg was used during the following 6 days. Control mice were administered with an equal volume of PBS. Five randomly selected mice in each of the groups were euthanized on days 5 and 7 post infection, respectively. BALF and lung tissue samples were collected from these mice for pathologic, immunologic and virologic assays.

### Cell intervention

Pre-treated MLE12 cells with rapamycin (100 nM) (Cell Signaling Technology, MA) for 1 h, were re-stimulated with H1N1 virus at multiplicity of infection (MOI) = 0.01 for 2 h and co-cultured with rapamycin (100 nM) with or without oseltamivir carboxylate (10 μg/ml) for 22 hours. After 24 h of culture, cells were harvested for analyzing the protein expressions.

### Measurement of viral RNA copies from cell-free supernatant

The MLE12 cells were pretreated with or without rapamycin (100nM) for 1 h. After washing with PBS, cells were infected with pH1N1 (MOI = 0.01) for 2 h. The infected cells were washed with PBS and incubated with rapamycin (100 nM) with or without oseltamivir carboxylate (10 μg/ml). Cell-free supernatants were harvested at the indicated hpi. Viral RNA was extracted from the supernatants (100 μl) using RNeasy Mini Kit (QIAGEN, Germany) following the manufacturer's instructions. Viral matrix (M) genes were measured by the QuantiTect Probe RT-PCR Kit (QIAGEN, Germany).

The following primer and probe were used: forward, 5’- GACCRATCCTGTCACCTCTGAC-3’, reverse, 5’-AGGGCATTYTGGACAAAKCGTCTA-3’, probe, 5’-FAM-TGCAGTCCT CGCTCACTGGGCACG-BHQ1-3’.

### Hematoxylin and eosin (H&E) staining

The lung samples were immediately fixed in 10% formalin, embedded in paraffin, and sectioned (4μm thickness) for histopathologic analysis. The sections were stained with hematoxylin and eosin (H&E) for examination by light microscopy.

### Lung injury score

Slides were randomized, read blindly, and then scored using a semiquantitative scoring system as described previously [76]. Edema, alveolar and interstitial inflammation, alveolar and interstitial hemorrhage, atelectasis, necrosis, and hyaline membrane formation were each scored on a 0-to 4-point scale: no injury = score of 0; injury in 25% of the field = score of 1; injury in 50% of the field = score of 2; injury in 75% of the field = score of 3; and injury throughout the field = score of 4. Results were confirmed by an experienced and qualified pathologist.

### Infiltration score

Each slide were assessed the degree of inflammatory cell infiltration of the main bronchus and the surrounding three large vessels, the mean values were obtained [77]. The criteria were no inflammatory cells = score of 0, a few inflammatory cells = score of 1, more uneven distribution of inflammatory cells = score of 2, a large number of inflammatory cells in relatively uniform distribution and rare gathered into a group = score of 3, a large number of inflammatory cells clump = score of 4. Results were confirmed by an experienced and qualified pathologist.

### Immunohistochemical staining

Paraffin-embedded lung sections were deparaffinized with xylene and hydrated using graded alcohols. The mTORC1 activity was assessed using anti-p-mTOR antibody (1:800, Abcam, Cambridge, UK) and anti-p-S6RP antibody (1:200, Cell Signaling Technology, MA). Macrophage infiltration was detected using anti-CD68 antibody (1:200, Abcam, Cambridge, UK) [78, 79]. The antibody was then detected using a standard streptavidin-biotin detection system (Beijing Zhongshan Biotechnology Co., Ltd., Beijing, China) according to the manufacturer’s instructions.

### Viral titer determination

The virus titrations were performed by end-point titration in MDCK cells. Lung samples were homogenized in 1 ml of sterile PBS. MDCK cells were inoculated with tenfold serial dilutions of homogenized lung tissues in 96-well plates. At 1 h post infection, cells were washed once with PBS and incubated in 200 μl of infection medium, consisting of EMEM, 100 μg/ml streptomycin, 100 IU/ml penicillin and 1 μg/ml TPCK-trypsin (Gibco, Life Technologies, NY). At 3 dpi, supernatants of infected cell cultures were tested for agglutinating activity using turkey erythrocytes as an indicator of cellular infection. Infectious titers were calculated from five replicates using the Reed-Muench method [80].

### Myeloperoxidase (MPO) and reactive oxygen species (ROS) assay

Lung samples were homogenized in 1 ml of sterile PBS and the supernatants were collected. The activity of MPO was measured using myeloperoxidase assay kit (Nanjing jiancheng bioengineering institute, Jiangsu, China). The activity of ROS was measured using ELISA kit (Bio swamp, Hubei, China) according to the manufacturer’s instructions.

### Analysis of BALF

The lungs were lavaged with 4 × 0.5 ml of PBS. The BALF was centrifuged 207 g at 4°C for 10 minutes, and the supernatant was collected and stored at -80°C for further analysis. The cells were resuspended in 1 ml of PBS and the total cell numbers were counted with a hemocytometer. Cytospin samples were prepared by centrifuging the suspensions at 350 rpm for 10 minutes using Shandon Cytospin 4 (Thermo scientific, Cheshire, UK) and cell differentials were determined by counting at least 300 leukocytes on Giemsa stain according to manufacturer’s instructions.

### Lactate dehydrogenase (LDH) assay

LDH activity in BALF was measured using lactate dehydrogenase assay kit (Nanjing jiancheng bioengineering institute, Jiangsu, China).

### Cytokine and chemokine analysis

The IL-1β, eotaxin, IFN-γ, IL-1α, IL-1α, IL-10, KC, MCP-1, MIP-1β, RANTES, TNF-α, G-CSF, GM-CSF, IL-6, IL-12 (p40), IL-12 (p70), IL-17 and MIP-1α levels in BALF were measured by Bio-Plex Mouse Cytokine Panel Assay Kit (Bio-Rad Laboratories, CA) according to the manufacturer’s instructions. The IL-18 level in BALF was measured by ELISA Kit (Mouse IL-18 ELISA KIT, Abcam, Cambridge, UK).

### Real-time PCR

Total RNA was extracted using the RNeasy Mini kit (Qiagen, Germany), and complementary DNA was synthesized with the PrimeScript RT Master Mix (Perfect Real Time) (TaKaRa, China). Real-time PCR (RT-PCR) was performed using the SYBR Premix Ex Taq II (Tli RNaseH Plus) (TaKaRa, China).

The following primers were used: NLRP3, forward: 5’-AATGCTGCTTCGACATCTCC-3’, reverse: 5’-CCAATGCGAGATCCTGACAA-3’; ASC, forward: 5’-TGAGCAGCTGCAAACGACTA-3’, reverse: 5’-CACGAACTGCCTGGTACTGT-3’; Caspase-1, forward: 5’-GTGGAGAGAAACAAGGAGTGG-3’, reverse: 5’-AATGAAAAGTGAGCCCCTGAC-3’; IL-1β, forward: 5’-GGTGTGTGACGTTCCCATTA-3’, reverse: 5’-GGCCACAGGTATTTTGTCGT-3’; IL-18, forward: 5’-ACAGGCCTGACATCTTCTGC-3’, reverse: 5’-CCTTGAAGTTGACGCAAGAGT-3’; GAPDH, forward: 5’-ATTCCACCCATGGCAAATTC-3’, reverse: 5’-CGCTCCTGGAAGATGGTGAT-3’.

### Western blot analysis

Protein samples were extracted from lung tissues and MLE12 cells. Proteins were separated by 10% SDS-polyacrylamide gel electrophoresis. After electrophoresis, the proteins were transferred to PVDF membranes by the wet transfer method. Each membrane was blocked with TBST with 5% non-fat dried milk for 1 hour at room temperature, and then incubated overnight at 4°C with primary antibodies directed against mTOR (1:1000, Cell Signaling Technology, MA), p-mTOR (1:500, Cell Signaling Technology, MA), S6K (1:1000, Cell Signaling Technology, MA), p-S6K (1:500, Cell Signaling Technology, MA), S6RP (1:1000, Cell Signaling Technology, MA), p-S6RP (1:500, Cell Signaling Technology, MA), NLRP3 (1:500, Adipogen, CA), ASC (1:1000, Cell Signaling Technology, MA), pro-caspase-1 (1:1000, Adipogen, CA), caspase-1 p20 (1:500, Adipogen, CA), pro-IL-1β (1:1000, Abcam, Cambridge, UK), IL-1β (1:500, Abcam, Cambridge, UK), p65 (1:1000, Cell Signaling Technology, MA), p-p65 (1:500, Cell Signaling Technology, MA), IκB (1:2000, Cell Signaling Technology, MA), p-IκB (1:1000, Cell Signaling Technology, MA), SQSTM1/p62 (1:1000, Cell Signaling Technology, MA), Beclin (1:1000, Cell Signaling Technology, MA), LC3 I/II (1:500, Cell Signaling Technology, MA) and β-actin (1:5000, Abcam, Cambridge, UK). The appropriate HRP-coupled secondary antibody was added and incubated for 1 hour at room temperature. The membranes were then treated with enhanced chemiluminescence western blot detection reagents (Millipore corporation, MA), and the binding of specific antibodies was detected by chemiluminescence.

### Statistical analysis

Survival curves were generated by the Kaplan-Meier method and statistical analyses were performed using the log-rank test. The statistical significance between two groups was assessed by Student’s t-tests. For comparisons between ≥ 3 groups, analysis was done by one-way ANOVA. A two-sided p value < 0.05 was considered statistically significant.

### Ethics statement

The experiments were performed in biosafety level 3 facilities in compliance with governmental and institutional guidelines. This study was carried out in accordance with the recommendations of the Chinese National Guidelines for the Care of Laboratory Animals and the Institutional Animal Care and Use Committee of the Institute of Laboratory Animal Science, Peking Union Medical College [81]. The protocol was approved by the Institutional Animal Care and Use Committee (ILAS-PC-2015-016).

## Supporting information

S1 Fig mTOR signaling pathway was activated after pH1N1 infection. (A) Western blot analysis of p-mTOR, mTOR, p-S6K, S6K, p-S6RP, and S6RP expression in the lungs of control and infected mice at 5 dpi. Representative immunohistochemical images of p-mTOR (B) and p-S6RP (C) expression in the bronchiolar epithelium and other severely inflamed lung tissues at 5 dpi. Scale bar = 200 or 100 μm, original magnification: ×100 or ×200. Data are representative of two independent experiments (n = 3 for each group).
(TIF)

S2 Fig Oseltamivir and rapamycin could inhibit viral spread *in vitro*. The MLE12 cells were infected with pH1N1 (MOI = 0.01) for 2 h. After washing with PBS, the cell-free supernatants in different treatment groups were collected to detect copies of viral RNA by RT-PCR at 24 hpi. Data are representative of two independent experiments and presented as mean ± SEM. * and ** represent *p* < 0.05 and 0.01, respectively.
(TIF)

S3 Fig Rapamycin can directly modulate the host immune response. Uninfected MLE12 cells were co-incubated with oseltamivir carboxylate (10 μg/ml), rapamycin (100 nM) or oseltamivir carboxylate (10 μg/ml) plus rapamycin (100 nM). The cells were harvested and analyzed for NLRP3, IL-1β and pro-IL-1β protein expression by western blotting after 24 hours (A). (B) Expression of NLRP3 relative to β-actin. (C) Expression of IL-1β and pro-IL-1β relative to β-actin. Data are representative of two independent experiments and presented as mean ± SEM. *, ** and *** represent *p* < 0.05, 0.01 and 0.001, respectively.
(TIF)

S4 Fig Rapamycin induced autophagy *in vitro*. The MLE12 cells were infected with pH1N1 (MOI = 0.01) for 2 h. After washing with PBS, cells were incubated with oseltamivir carboxylate (10 μg/ml), rapamycin (100 nM) or oseltamivir carboxylate (10 μg/ml) plus rapamycin (100 nM). The cells were harvested and analyzed for protein expression by western blotting at indicated time. (A) Protein levels of SQSTM1/p62 and LC3 I/II at 24 hpi. (B) Expression of SQSTM1/p62, LC3 I and LC3 II relative to β-actin, and LC3 II relative to LC3 I. (C) Protein levels of SQSTM1/p62 and Beclin at 48 hpi. (D) Expression of SQSTM1/p62 and Beclin relative to β-actin. (E) Protein levels of SQSTM1/p62 and Beclin at 72 hpi. (F) Expression of SQSTM1/p62 and Beclin relative to β-actin. Data are representative of two independent experiments and presented as mean ± SEM. * and ** represent *p* < 0.05 and 0.01, respectively.
(TIF)
