# Supplementary figures and images for: Delayed oseltamivir plus sirolimus treatment attenuates H1N1 virus-induced severe lung injury correlated with repressed NLRP3 inflammasome activation and inflammatory cell infiltration
Source: PLoS Pathog. 2018 Nov 13;14(11):e1007428. doi: 10.1371/journal.ppat.1007428 (PMC6258564; doi:10.1371/journal.ppat.1007428)

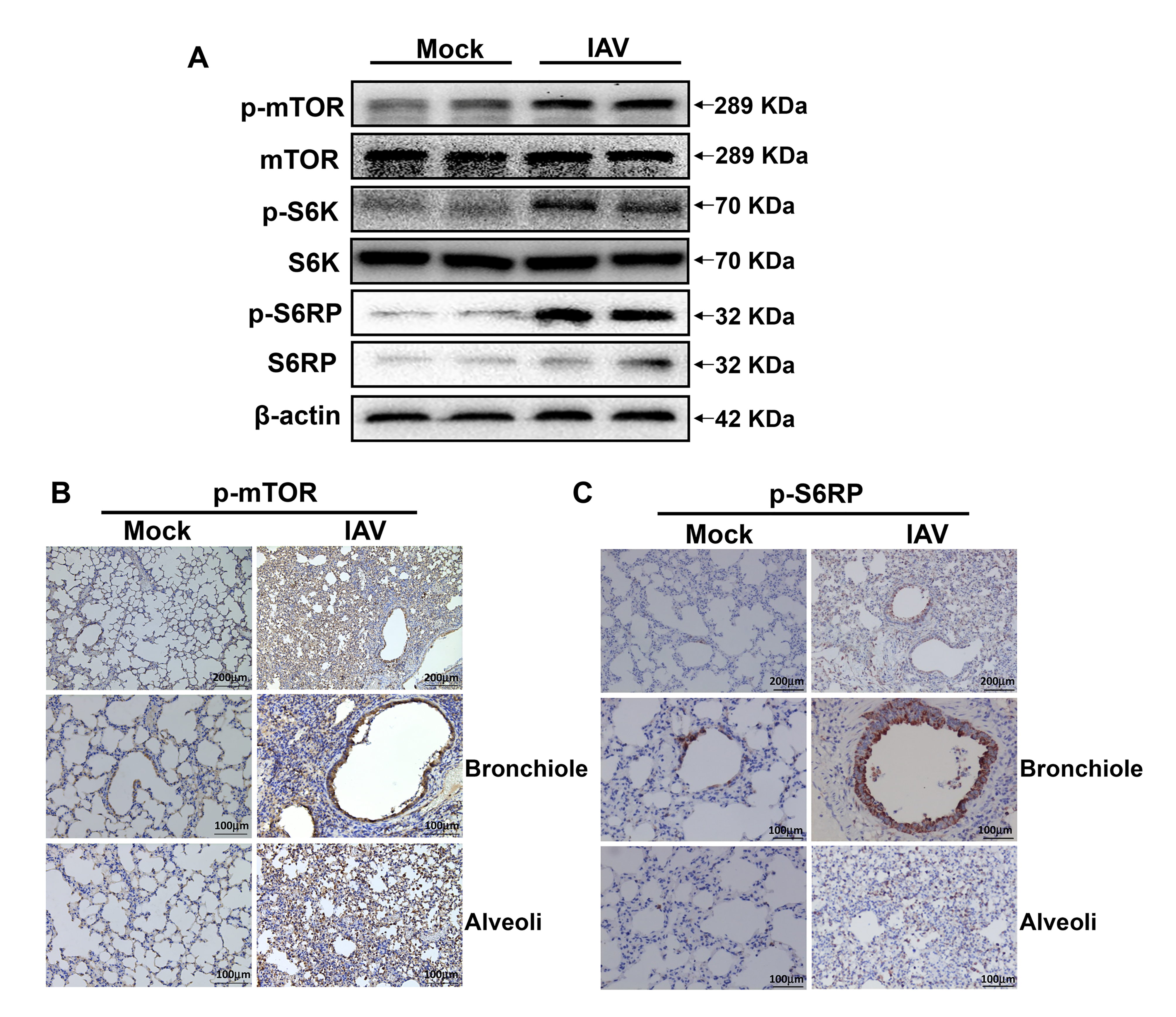

Supplement: S1 Fig — (A) Western blot analysis of p-mTOR, mTOR, p-S6K, S6K, p-S6RP, and S6RP expression in the lungs of control and infected mice at 5 dpi. Representative immunohistochemical images of p-mTOR (B) and p-S6RP (C) expression in the bronchiolar epithelium and other severely inflamed lung tissues at 5 dpi. Scale bar = 200 or 100 μm, original magnification: ×100 or ×200. Data are representative of two independent experiments (n = 3 for each group). (TIF) [file ppat.1007428.s001.tif]

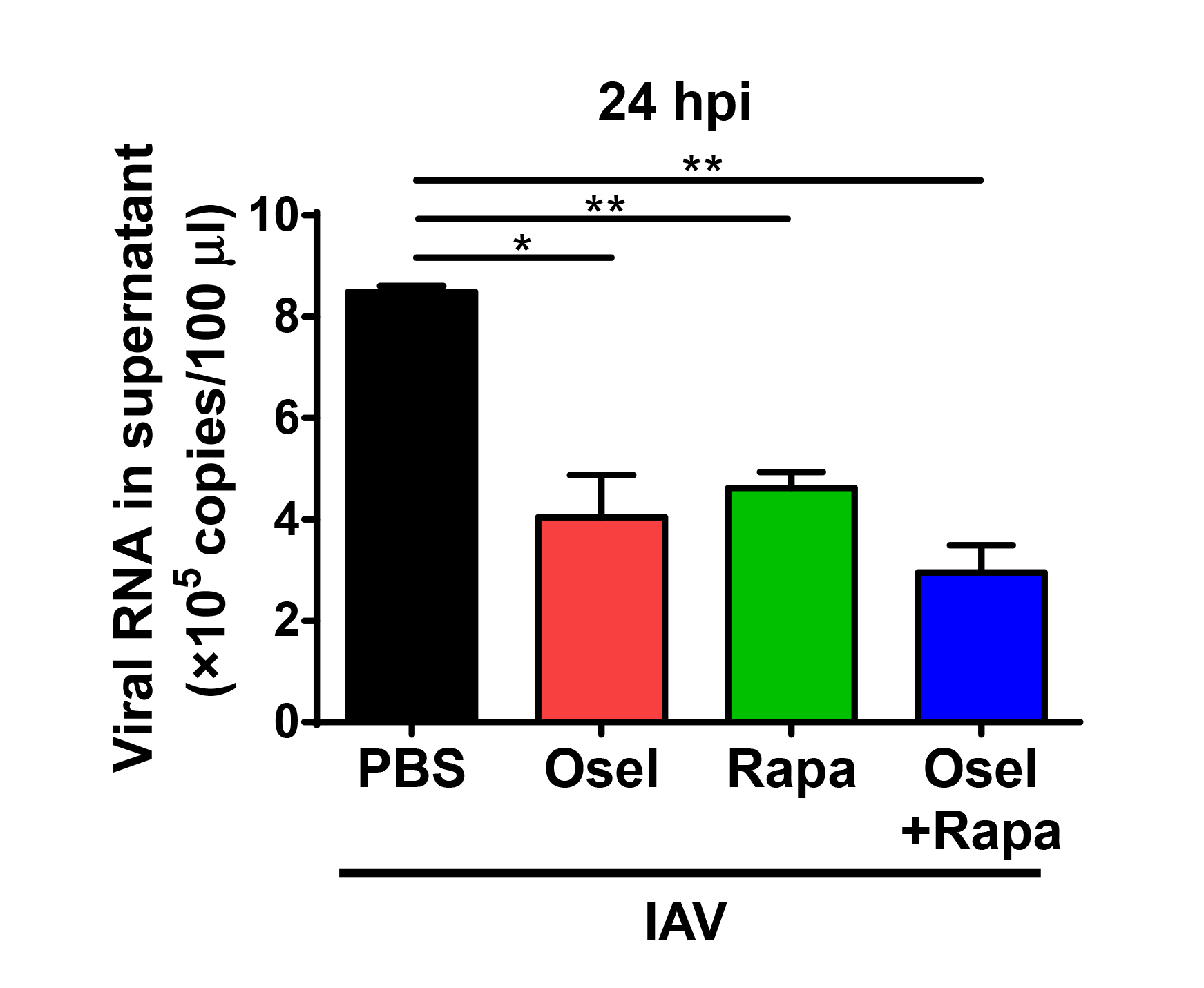

Supplement: S2 Fig — The MLE12 cells were infected with pH1N1 (MOI = 0.01) for 2 h. After washing with PBS, the cell-free supernatants in different treatment groups were collected to detect copies of viral RNA by RT-PCR at 24 hpi. Data are representative of two independent experiments and presented as mean ± SEM. * and ** represent p < 0.05 and 0.01, respectively. (TIF) [file ppat.1007428.s002.tif]

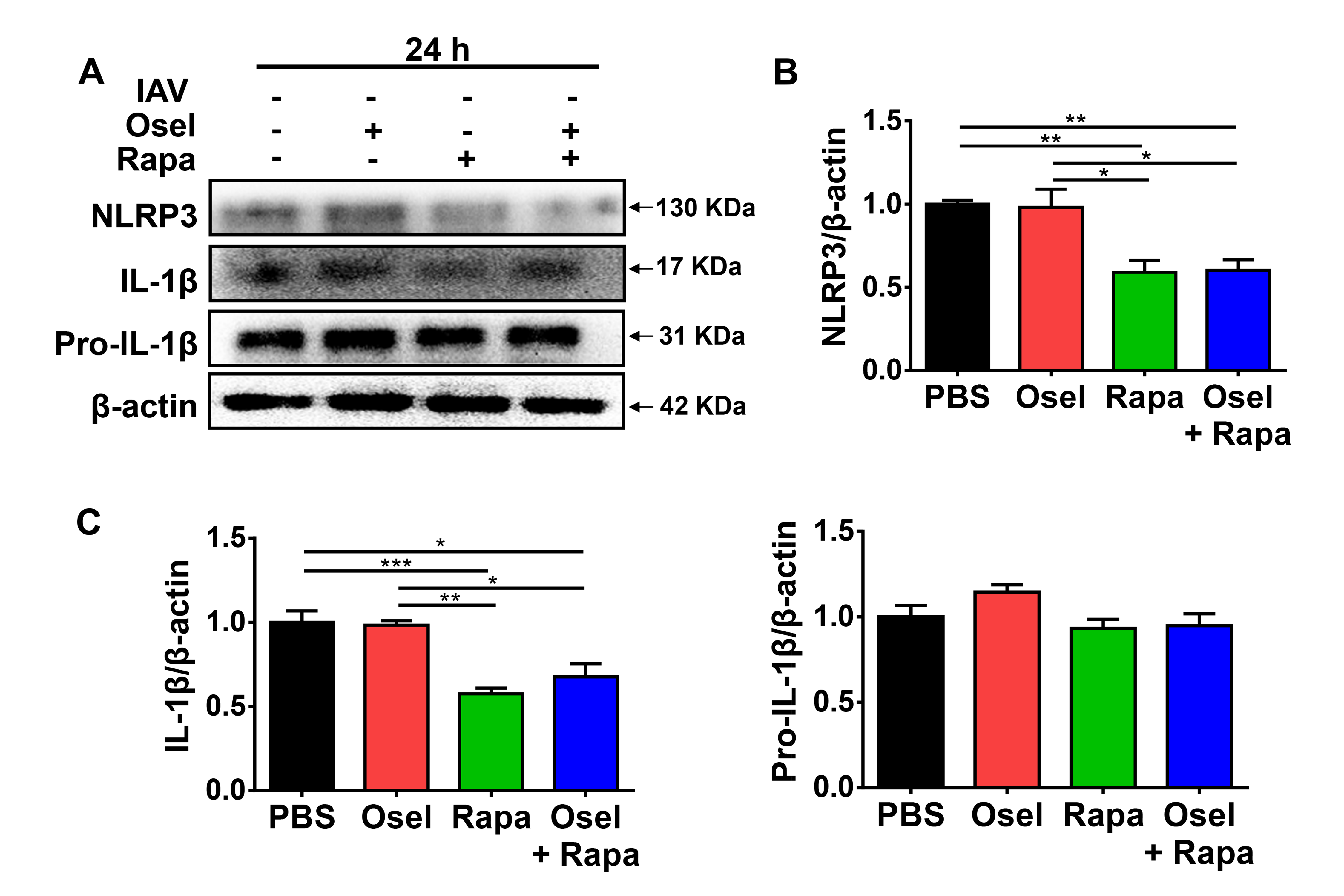

Supplement: S3 Fig — Uninfected MLE12 cells were co-incubated with oseltamivir carboxylate (10 μg/ml), rapamycin (100 nM) or oseltamivir carboxylate (10 μg/ml) plus rapamycin (100 nM). The cells were harvested and analyzed for NLRP3, IL-1β and pro-IL-1β protein expression by western blotting after 24 hours (A). (B) Expression of NLRP3 relative to β-actin. (C) Expression of IL-1β and pro-IL-1β relative to β-actin. Data are representative of two independent experiments and presented as mean ± SEM. *, ** and *** represent p < 0.05, 0.01 and 0.001, respectively. (TIF) [file ppat.1007428.s003.tif]

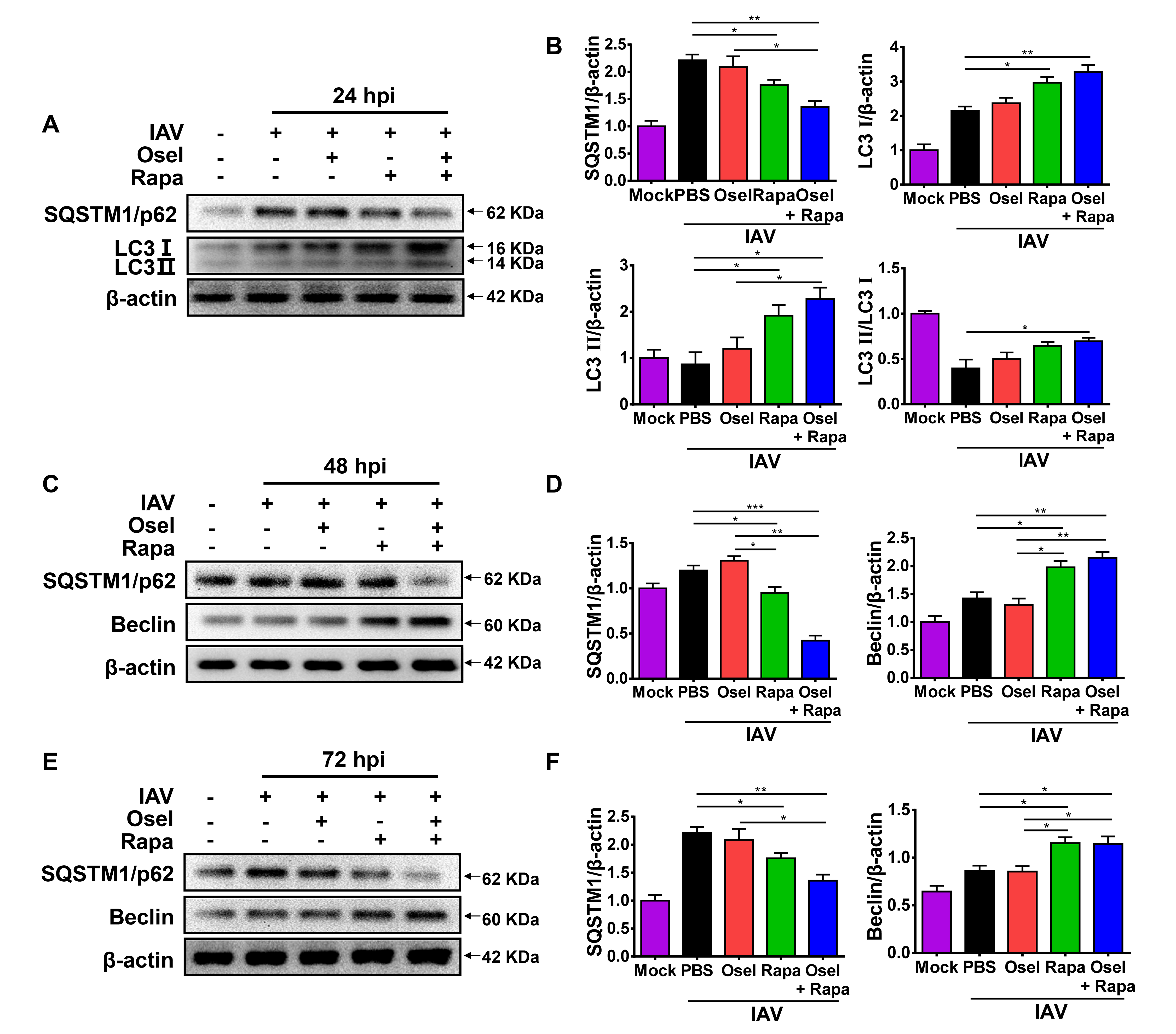

Supplement: S4 Fig — The MLE12 cells were infected with pH1N1 (MOI = 0.01) for 2 h. After washing with PBS, cells were incubated with oseltamivir carboxylate (10 μg/ml), rapamycin (100 nM) or oseltamivir carboxylate (10 μg/ml) plus rapamycin (100 nM). The cells were harvested and analyzed for protein expression by western blotting at indicated time. (A) Protein levels of SQSTM1/p62 and LC3 I/II at 24 hpi. (B) Expression of SQSTM1/p62, LC3 I and LC3 II relative to β-actin, and LC3 II relative to LC3 I. (C) Protein levels of SQSTM1/p62 and Beclin at 48 hpi. (D) Expression of SQSTM1/p62 and Beclin relative to β-actin. (E) Protein levels of SQSTM1/p62 and Beclin at 72 hpi. (F) Expression of SQSTM1/p62 and Beclin relative to β-actin. Data are representative of two independent experiments and presented as mean ± SEM. * and ** represent p < 0.05 and 0.01, respectively. (TIF) [file ppat.1007428.s004.tif]
